# Epidemiology of COVID-19 Outbreak on Cruise Ship Quarantined at Yokohama, Japan, February 2020

**DOI:** 10.3201/eid2611.201165

**Published:** 2020-11

**Authors:** Expert Taskforce for the COVID-19 Cruise Ship Outbreak

**Affiliations:** National Institute for Infectious Diseases, Shinjuku-ku, Tokyo, Japan

**Keywords:** COVID-19, epidemiology, quarantine, cruise ship, Japan, 2019 novel coronavirus disease, severe acute respiratory syndrome coronavirus 2, zoonoses, coronavirus disease, SARS-CoV-2, viruses

## Abstract

To improve understanding of coronavirus disease (COVID-19), we assessed the epidemiology of an outbreak on a cruise ship, February 5–24, 2020. The study population included persons on board on February 3 (2,666 passengers, 1,045 crew). Passengers had a mean age of 66.1 years and were 55% female; crew had a mean age of 36.6 years and were 81% male. Of passengers, 544 (20.4%) were infected, 314 (57.7%) asymptomatic. Attack rates were highest in 4-person cabins (30.0%; n = 18). Of crew, 143 (13.7%) were infected, 64 (44.8%) asymptomatic. Passenger cases peaked February 7, and 35 had onset before quarantine. Crew cases peaked on February 11 and 13. The median serial interval between cases in the same cabin was 2 days. This study shows that severe acute respiratory syndrome coronavirus 2 is infectious in closed settings, that subclinical infection is common, and that close contact is key for transmission.

On January 30, 2020, the director-general of the World Health Organization (WHO) declared the outbreak of a novel coronavirus disease (COVID-19) a public health emergency of international concern (*1*). Three days later, health authorities in Hong Kong alerted health authorities in Japan that a COVID-19 case was confirmed in Hong Kong on February 1 in a patient who had developed symptoms on January 19 and disembarked from a cruise ship en route to Yokohama, Japan, on January 25. The ship had initiated its voyage from Yokohama on January 20 and visited ports in Japan, Hong Kong, Vietnam, and Taiwan before returning to Yokohama.

Authorities in Japan ordered the captain of the ship to remain at Yokohama port upon arrival, with no persons allowed to disembark. At that time, 2,666 passengers and 1,045 crew members were on board, totaling 3,711 persons. On February 3, health authorities in Japan reviewed logs at the onboard clinic for symptomatic (febrile or respiratory) patients and obtained respiratory specimens from them. On February 5, severe acute respiratory syndrome coronavirus 2 (SARS-CoV-2) was detected through real-time reverse transcription PCR (rRT-PCR) in 1 of these specimens.

At 7:00 am on February 5, all persons on board were ordered to remain in their cabins for 14 days and were informed that this period could be extended if they had had close contact with someone who had a confirmed case. As of March 8, a total of 696 COVID-19 cases had been reported from this ship (*2*). Our objective was to characterize the epidemiology of this cruise ship outbreak to improve global understanding of COVID-19 and inform response measures for the global outbreak.

## Design and Methods

We used descriptive and analytical statistics to conduct this epidemiologic assessment of quarantine measures aboard the ship during February 5–24, 2020, including all persons aboard the ship during that time. The assessment was approved by the institutional review board at the National Institute of Infectious Diseases, Tokyo.

Persons on the ship included all 3,711 persons (crew and passengers) registered with the cruise ship owner as being on board on February 3, 2020, when the ship arrived at Yokohama. Beginning on February 5, passengers were quarantined in their cabins with their cabinmates. Passenger cabin capacity ranged from 1 to 4 persons. Passengers, organized by deck and section, were allowed a 60-minute period on an exterior deck each day, during which they were instructed to wear masks, refrain from touching anything, and maintain a 1-meter distance from others. Monitors observed these periods. After each group came a 30-minute period in which the areas were disinfected. Room cleaning was suspended. Food and clean linens were delivered to cabin doors by crew, and dirty dishes and linens were picked up at cabin doors by crew. Clean services and dirty services were performed by separate groups of the crew.

Crew members were ordered to remain in their cabins except to perform essential duties. Depending on the job and rank, crew cabin capacity ranged from 1 to 4 persons. Health authorities from Japan distributed personal protective equipment to crew members and instructed them on proper use.

On February 7, authorities provided thermometers to passengers and instructed them to check body temperature regularly. Thermometers were also distributed to the crew, although not to every member, and crew members were requested to monitor body temperature. A fever call center was established inside the ship; persons on board were asked to call the center if they had a temperature >37.5°C. A respiratory specimen was obtained from any person with fever or respiratory symptoms. Specimens were tested for SARS-CoV-2 by rRT-PCR (*3*). Any person with a positive rRT-PCR specimen was defined as having COVID-19; these persons disembarked and were transferred to an isolation facility in Japan.

Cabinmates of confirmed case-patients were classified as close contacts, and their 14-day observation period was reset on the last day of contact with the case-patient. Any close contact with a positive test result was considered a case-patient; these persons disembarked and were taken to an isolation facility. A negative test result allowed the person to remain on board in quarantine. Because of the limited availability of laboratories with the capacity to test for SARS-CoV-2 early in this outbreak, only symptomatic close contacts were tested initially. As laboratory capacity in Japan increased, the testing strategy changed to include, first, all contacts of case-patients, then all passengers (beginning with older age groups), and then all crew. Any person on board who developed serious illness, including non–COVID-19 conditions (such as myocardial infarction), was taken off the ship, sent to a healthcare facility, and tested for SARS-CoV-2. To be released from quarantine, a person had to complete a 14-day observation period without being defined as a close contact during the period, pass medical screening for fever and respiratory symptoms at the end of the 14-day period, and obtain >1 negative test result (and no positive result) during the 14-day period.

Primary data sources for this article included rRT-PCR results, information from the ship manifest (age, sex, country of passport, cabin number, and classification as passenger or crew), and symptom data (presence or absence of symptoms, onset date, and severe outcomes) recorded at the time of respiratory specimen collection or through standard public health follow-up of cases. A confirmed case of COVID-19 was defined as an illness in any person on board the ship during the study period who had 1 positive rRT-PCR result for SARS-CoV-2, independent of symptoms. A symptomatic case was defined as one with the presence of COVID-19 related symptoms, such as fever or cough, at the time of respiratory specimen collection.

We classified countries of origin according to the passport country listed in the ship manifest. We then used number of cases reported to WHO as of February 5 (*4*) to group countries as having 0 cases reported, 1–5 cases, or >5 cases reported.

We calculated descriptive statistics and used denominators based on the ship manifest as of February 5. Because of the different natures of the quarantines for passengers and crew, we analyzed them, for most analyses, as separate populations. A series interval of 4 days (Nishiura HL, unpub. data, https://doi.org/10.1101/2020.02.03.20019497) was used to assign secondary or tertiary case status within cabins: secondary cases had to have onset dates >4 days after primary cases, and tertiary cases had onset days >4 days after secondary cases. 

To conduct spatial analysis, we analyzed the spatial distribution of confirmed cases across decks. We used the assigned cabin numbers of passengers and crew members for mapping. We used the date of illness onset, if known. If the date of onset was unknown or the patient was asymptomatic, we subtracted the mean delay from illness onset to diagnosis (3 days) from the date of confirmation and set it this as the date of illness onset. To calculate attack rates by cabin occupancy, we removed from the analyses 110 passengers who stayed in crew cabins. 

This assessment focused on providing additional epidemiologic characteristics of COVID-19, a new infectious disease with high public health risk. It was granted institutional review board approval with the use of simplified informed consent procedures at the National Institute of Infectious Diseases.

## Results

The study population included 2,666 passengers and 1,045 crew members. The mean age of passengers was 66.1 years, and 55% were female. Most (84%) passengers’ country of origin had reported >5 COVID-19 cases by February 5. The mean age of the crew was 36.6 years, and 19% were female. Most crew (69%) had countries of origin that had reported 1–5 COVID-19 cases as of February 5, and 19% of crew members’ countries of origin had reported zero cases.

During the study period, 544 (20.4%) of the passengers were defined as case-patients (Table 1). Passenger case-patients averaged 67.9 years of age (SD +12.0). Among case-patients, 314 (57.7%) were asymptomatic, 33 had nonfatal severe outcomes, and 7 died. Attack rates among passengers were highest among those who stayed in 4-person cabins (30.0%; n = 18), followed by 3-person cabins (22.0%; n = 27), 2-person cabins (20.6%; n = 491), and 1-person cabins (8%; n = 6).

**Table 1:** Characteristics of persons aboard quarantined cruise ship, Japan, February 5–24, 2020

Characteristic	No. (%) persons

Passengers, n = 2,666			Crew, n = 1,045	
Cases, n = 544; 20.4%	Noncases, n = 2,122; 79.6%		Cases, n = 143; 13.7%	Noncases, n = 902; 86.3%
Age group					
0–9	1 (6.3)	15 (93.8)		0	0
10–19	5 (22.7)	17 (77.3)		0	1 (100)
20–29	9 (18.4)	40 (81.6)		29 (9.7)	269 (90.3)
30–39	7 (11.9)	52 (88.1)		55 (14.9)	315 (85.1)
40–49	4 (5.5)	69 (94.5)		41 (15.8)	219 (84.2)
50–59	49 (16.2)	253 (83.8)		17 (17.3)	81 (82.7)
60–69	177 (19.4)	734 (80.6)		1 (5.6)	17 (94.4)
70–79	239 (23.7)	769 (76.3)		0	0
80–89	51 (23.7)	164 (76.3)		0	0
90–99	2 (18.2)	9 (81.8)		0	0
Sex					
M	245 (20.6)	944 (79.4)		112 (13.3)	731 (86.7)
F	299 (20.2)	1,178 (79.8)		31 (15.3)	171 (84.7)
No. cases reported from country of origin as of Feb 5					
0	11 (15.1)	62 (84.9)		27 (13.5)	173 (86.5)
1–5	62 (17.5)	293 (82.5)		10 (13.9)	617 (86.1)
>5	471 (21.0)	1767 (79.0)		16 (12.5)	112 (87.5)

Among crew, 143 (13.7%) were defined as case-patients. Crew case-patients averaged 37.7 years of age (SD +9.0). Among these, 64 (44.8%) were asymptomatic; none had fatal or nonfatal severe outcomes.

**Figure 1:**
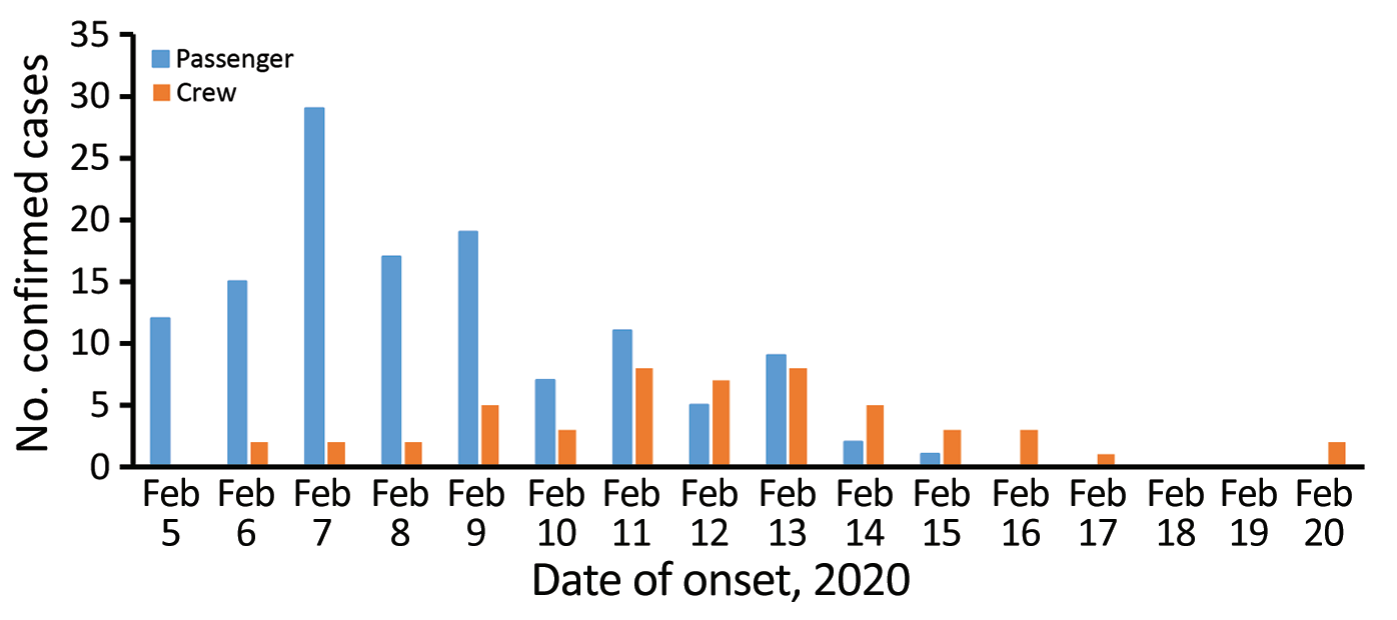
Number of coronavirus disease cases aboard cruise ship quarantined in Japan, by date of symptom onset, February 5–20, 2020 (n = 178). The graph displays the number of cases by symptom onset date for the cases for which onset date was available for passengers and crew members. Cases without a recorded symptom onset date were excluded.

Figure 1 displays the number of cases by date of onset for the study period for cases with an available onset date (in 127 passengers and 51 crew). Symptomatic cases among passengers peaked on February 7. Another 35 passenger cases had onset dates before February 5. Cases among crew peaked on February 11 and 13.

**Figure 2:**
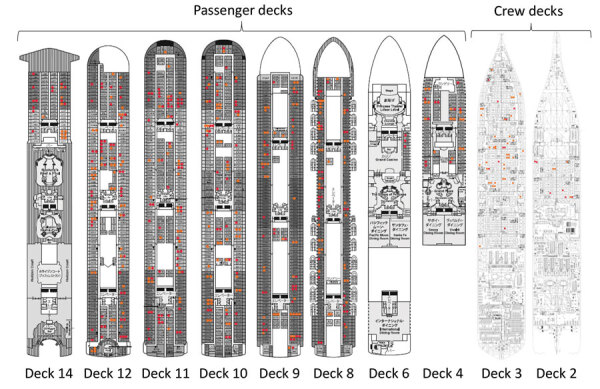
Spatial mapping of coronavirus disease cases aboard cruise ship quarantined in Japan, by deck, February 3–25, 2020. The ship had 18 decks (i.e., floors). Decks 4–14 contained cabin rooms for passengers; decks 2 and 3 were for crew. Red dots indicate the latest cases in cabins (i.e., cases with illness onset starting February 13–16 or infected but asymptomatic persons whose samples tested positive by real-time reverse transcription PCR from February 16 onward. Orange dots represent cases in passengers or crew who appeared to be infected before those periods.

Figure 2 shows the spatial snapshot of COVID-19 cases during February 13–16, 2020. Infected passengers were observed across different decks, and there was no identifiable aggregation or large-scale clustering by deck or zone. Crew decks produced a diffusive pattern, although a large number of cases was observed among restaurant staff on deck 3.

Table 2 presents cases among passengers and crew according to symptom presentation. For passengers, there were more asymptomatic than symptomatic cases for all age groups except for those 20–29 and 80–89 years of age. For crew, higher proportions of symptomatic cases were observed among all age groups except those 40–49 years of age, for which they were similar.

**Table 2:** Symptomatic and asymptomatic cases among passengers and crew aboard quarantined cruise ship, by age group, Japan

Age group, y	No. (%) persons				
	Passengers			Crew	
	Symptomatic	Asymptomatic		Symptomatic	Asymptomatic
0–9	0	1 (100)		0	0
10–19	2 (40.0)	3 (60.0)		0	0
20–29	7 (77.8)	2 (22.2)		18(62.1)	11(37.9)
30–39	2 (28.6)	5 (71.4)		30(54.5)	25(45.5)
40–49	1 (25.0)	3 (75.0)		20(48.8)	21(51.2)
50–59	19 (38.8)	30 (61.2)		11(64.7)	6(35.3)
60–69	75 (42.4)	102 (57.6)		0	1(100)
70–79	95 (39.7)	144 (60.3)		0	0
80–89	27 (52.9)	24 (47.1)		0	0
90–99	2 (100)	0		0	0
Total	230 (42.3)	314 (57.7)		79 (55.2)	64 (44.8)

Among 26 pairs of passenger cases that occurred in the same cabin, 9 (35%) had a serial interval of 5 days or greater. Three of these occurred in 3- or 4-person cabins. The median interval for these cases was 2 days (range 0–25 days; interquartile range 2–4 days). In the two 4-person cabins in which multiple cases were reported, 1 had serial intervals of 1, 3, and 0 days between the first and second, second and third, and third and fourth cases. The other had intervals of 5 days between the first and second and 6 days between second and third cases. Among crew members, 6 pairs of cases were identified in the same cabins, with serial intervals of 0, 1, 2, 3, 4, and 5 days.

## Discussion

Over 20 days, 22% of a cruise ship population of 3,711 was detected to have been infected with SARS-CoV-2. More than half of all case-patients were asymptomatic at the time of respiratory specimen collection. Passenger cases typically had onset dates earlier than crew cases, and many cases had onset dates before the ship’s arrival in Yokohama. Infection by that time was spread across multiple decks without spatial clustering. Passengers 10–29 years of age had similar infection rates to those 60–99 years of age. Passengers were more likely to be infected than crew and more likely to present asymptomatically than crew. Attack rates increased with increasing cabin occupancy.

Recent modeling on controlling COVID-19 outbreaks with isolation alone has shown that isolation alone is insufficient, even if contract tracing reaches nearly 100%; thus, additional public health interventions would be needed to control and halt an outbreak (*5*). Because of the nature of the ship, individual isolation of passengers and crew was not possible, and some crew members were required to continue essential services and ship functions. Thus, the quarantine measures should not have been expected to stop transmission completely. Nevertheless, a modeling study has suggested that the basic reproduction rate (R_0_) during the outbreak aboard this ship was 4 times higher than that for Wuhan, China, during its outbreak and that 79% of the population aboard would have been infected if not for the intervention (*6*). A second modeling study suggested that the R_0_ early in the outbreak would have produced 1,514 cases, and that a reduction by 50% would have resulted in 758 total cases, which is higher than the observed number of cases on board.

As the models and our findings have pointed out, there was substantial infection on board the ship before arrival in Yokohama (*7*). The index case that was reported by Hong Kong authorities was more likely an indicator case; that is, the first detected case among many infected persons. Dates of onset for passengers included in this assessment stretch back to January 20. Onboard clinic fever data (Appendix Figure 1) similarly showed an increasing trend since the beginning of the voyage. Most early infections occurred among passengers; given that crew dates of onset peaked on February 11 and 13, it is reasonable to assume that transmission from passengers to crew occurred, on average, 7 days earlier, on February 4 or 6, just before and just after quarantine began.

Spatial clustering was not identified on a specific deck or zone, and transmission does not seem to have spread to neighboring cabins, implying that droplet or contact transmission to nearby cabins was not the major mode of infection. Risk of infection did increase with cabin occupancy, but a relatively small proportion of cases in the same cabin had >4 days between their onsets, implying a common source of infection. Beyond that, however, the major transmission routes might include a common source outside the cabin and aerosolized fomite or contact transmission across different deck levels. One review of human coronaviruses, which did not include SARS-CoV-2, has shown that they can persist on inanimate surfaces up to 9 days but can be inactivated by surface disinfection procedures within 1 minute (*8*). Given recent reports of outbreaks of COVID-19 in conferences, live music houses, and religious gatherings, a common source event is justified. Such an event in the days before the quarantine would explain both the spatial distribution and the temporal distribution of symptomatic cases.

A key finding of this assessment was the high proportion of cases with asymptomatic infection. No other study has identified such rates. A large study of COVID-19 cases reported 1% asymptomatic cases (*9*). Nevertheless, in this population, every person was tested; thus, it may be representative of the true picture of infection and transmission for COVID-19 (Appendix Figure 2). If such is the case, then the proportion of fatal and severe cases (1.0%) is much lower than that reported globally (*2*,*7*,*9**–**11*). It is noteworthy that two thirds of the ship population was >60 years of age. This finding also implies that, given the current number of fatal and severe cases globally, there is substantial underdetection or underreporting of infection.

As described elsewhere (*12*), it is difficult to know whether these cases were truly asymptomatic, presymptomatic, or postsymptomatic. We reclassified »30 asymptomatic cases as symptomatic after learning through follow-up that the case-patients had developed symptoms. Some authors have suggested that presymptomatic transmission is possible (*13*,*14*). A high proportion of asymptomatic cases will pose serious challenges to understanding and controlling the global outbreak. Contact tracing will struggle to find chains of transmission, infected persons will pass through entry and exit screening posts, and seemingly healthy persons may infect vulnerable ones.

Several key considerations limit the findings in our study. First, throughout the study period, persons on board permanently disembarked for many reasons: confirmed cases, medical emergencies, family members with medical emergencies, country-assisted repatriations, and completion of quarantine requirements. Thus, the true denominators shrank over time, which may result in overestimating the true proportion of infected persons. Second, initial data collection was limited by the emergency nature of the response. For many of the early cases, symptom onset dates were obtained retrospectively. The differential approach to obtaining these data may introduce bias in comparing the prequarantine and quarantine periods. Third, laboratory testing was initially limited to symptomatic cases and close contacts but then expanded to include all passengers and crew. Thus, asymptomatic infection early in the study period may have been underestimated if these asymptomatic case-patients cleared their viral loads before being tested. Fourth, we are aware of >9 persons who tested negative while on board the ship and positive after being released. It is difficult to know for certain if these results were caused by false negatives, false positives, infections developing between the 2 tests, or an increased viral load between the 2 tests. Nevertheless, we cannot rule out that some infections may have gone undetected, which would underestimate the true proportion of infected persons. Fifth, 89 asymptomatic passengers were repatriated by chartered flights before receiving rRT-PCR tests. Some of them were confirmed positive after repatriation, but we could not incorporate their results into this study. It is thus possible that our findings underestimate the true proportion of affected persons, though the difference is not expected to be large.

Moving forward, we propose 4 key steps. First, public health authorities should adapt their response strategies to reflect the high proportion of asymptomatic cases found here. Second, the scientific community should investigate transmission routes other than person-to-person droplets. Third, the scientific community should conduct serologic surveys and shedding experiments to understand the role of asymptomatic infection and pre- and postsymptomatic phases of symptomatic infection on transmission. Fourth, the international maritime community should begin dialogue with national governments (especially quarantine and health sectors) to discuss the feasibility of quarantining large passenger ships.

As of March 8, 2020, all passengers and crew had disembarked from the ship, which was sanitized afterward. Public health measures may have prevented >2,000 COVID-19 cases, and quarantine prevented case-patients from being released into the community. The findings from this complex emergency, in one of the few populations to have been completely tested by rRT-PCR for SARS-CoV-2, imply that the virus is highly infectious, especially in a closed environment, but that it results in a lower proportion of fatal cases than previously reported.

As of August 11, 2020, a total of 19,936,210 confirmed cases had been reported to WHO, and there have been 732,499 deaths worldwide (*15*). Additional research into asymptomatic transmission of SARS-CoV-2 is urgently needed to understand the effectiveness of current global strategies to stop the COVID-19 outbreak.

Appendix Additional information about outbreak of COVID-19 on a cruise ship quarantined in Japan.
