# Supplementary material for: Epidemiology of COVID-19 Outbreak on Cruise Ship Quarantined at Yokohama, Japan, February 2020
Source: Emerg Infect Dis. 2020 Nov;26(11):2591–7. doi: 10.3201/eid2611.201165 (PMC7588545; doi:10.3201/eid2611.201165)
Supplement: Appendix — Additional information about outbreak of COVID-19 on a cruise ship quarantined in Japan. [file 20-1165-Techapp-s1.pdf]

Article DOI: https://doi.org/10.3201/eid2611.201165

Epidemiology of COVID-19 Outbreak on Cruise Ship Quarantined at Yokohama, Japan, February 2020

Appendix

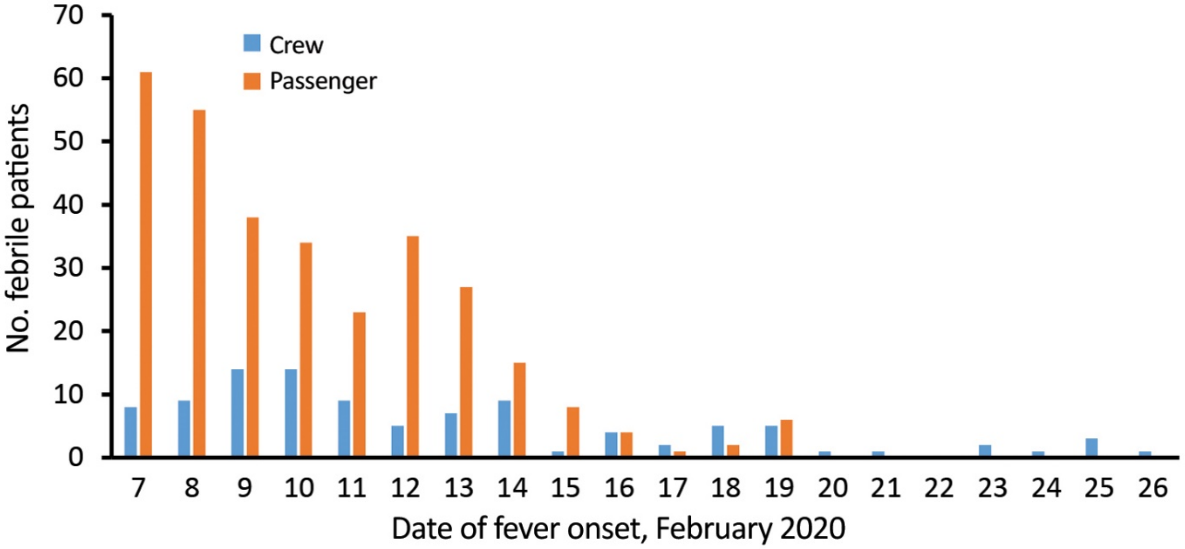

Appendix Figure 1. Number of febrile patients reporting to the fever call center on board the ship, February 7–26, 2020 (n = 410). Data are from the cruise ship's fever call center on the number of febrile patients, by passenger or crew member, February 7–26.

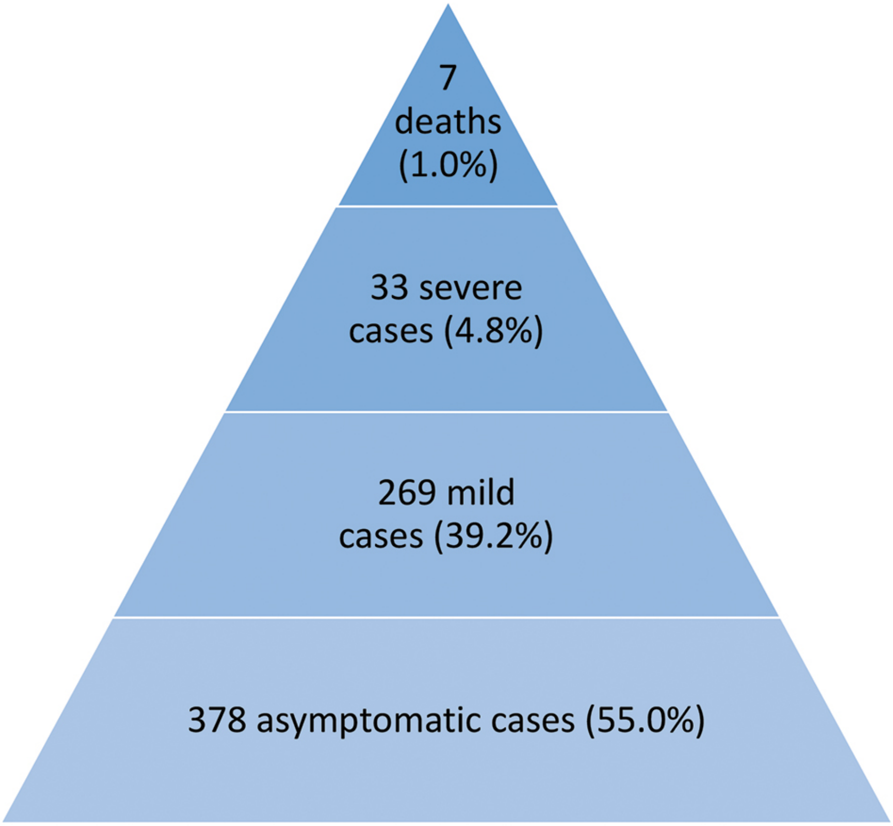

Appendix Figure 2. Proportion of fatal, severe, mild, and asymptomatic cases among a population in which all persons were tested by rRT-PCR for SARS-CoV-2. Because the entire population on board the cruise ship was tested with rRT-PCR for SARS-CoV-2, it was possible to illustrate these proportions.
